# Functional, antioxidant, and sensory properties of mixed‐fruit (pitaya, watermelon, and mint) and pitaya wines

**DOI:** 10.1002/fsn3.3334

**Published:** 2023-05-04

**Authors:** Tsung‐Ming Hu, Mohsen Gavahian, Rojina Pradhan, Si‐Yu Lu, Yung‐Lin Chu

**Affiliations:** ^1^ Department of Psychiatry Yuli Branch Taipei Veterans General Hospital Hualien Taiwan; ^2^ Department of Food Science College of Agriculture National Pingtung University of Science and Technology Pingtung Taiwan

**Keywords:** alcohol, antioxidant capacity, fermentation, mint, pitaya, watermelon

## Abstract

Fermentation of fruits offers a diverse range of flavors, smells, and colors. Colored fruits are rich in naturally occurring pigments, such as betacyanin. Hence, they are considered to possess powerful antioxidant activities. However, in wine production, such pigments often diversify the flavor and color of the wine. The objective of this study was to compare the quality of two types of wines: a single‐fruit (pitaya) wine and a mixed‐fruit wine that contains watermelon (*Citrullus lanatus*), mint (*Mintha spicata*), and pitaya (*Hylocereus costaricensis*). In this study, fresh pitaya, watermelon, and mint leaves were fermented using *Saccharomyces cerevisiae*. Juice extracts underwent fermentation at room temperature for 7 days under dark conditions. Physicochemical changes, such as pH, sugar content, specific gravity, and alcohol content, were observed daily. The antioxidant activities were measured by the 2,2‐diphenyl‐1‐picrylhydrazyl (DPPH) assay, the ferric reducing antioxidant power (FRAP) assay, and total phenolic contents (TPCs). After 14 days of fermentation, the alcohol contents of mixed and pitaya wine were 11.22% (v/v) and 11.25%, respectively. The total sugar content of the mixed wine was 8.0 °Brix, while that of pitaya wine was 7.0 °Brix. Moreover, pitaya wine exhibited a higher TPC (22.7 mg GAE/100 g D.W.), and better FRAP (3578 μmole/L) and DPPH scavenging ability (80.2%) compared to the mixed wine with a TPC of 21.4 mg GAE/100 g D.W., FRAP of 2528 μmole/L, and DPPH of 75.6%., while the addition of watermelon and mint did not change the alcohol percentage contents of wine.

## Graphical abstract

This is the first paper to demonstrate the anti‐oxidative ability and sensory evaluation in different types of pitaya wines.

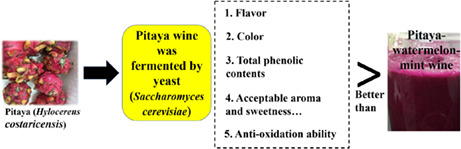

## 1 INTRODUCTION

Wine fermentation is the process of making alcohol from carefully selected fruits. During fermentation, the sugar present in the juice will react with the yeast to produce alcohol and carbon dioxide as a by‐product. Nowadays, colored and non‐colored wines (white wine) are popularly available all over the world because of their taste, color, and alcohol volume (Ali, 2018). Most of these popular wines are produced from grapes, as reported in the literature (Gavahian et al., 2022). However, grape production is largely restricted to climatic regions. Hence, there is ongoing research to find optional tropical and subtropical fruit sources that can produce similar or better wine.

The pitaya fruit, a Cactaceae family member, is native to Mexico and widely cultivated in tropical and subtropical areas. According to previous study, pitaya fruit is rich in vitamins, dietary fiber, betacyanin, organic acids, amino acids, and sugars. Studies suggest that pitaya betacyanins exhibit antidiabetic effects and modulate glycemic response (Nishikito et al., 2023). In this study, watermelon and mint were combined with pitaya to produce a novel tropical‐flavored wine. Mint is widely used in flavored wine (Picard, et al., 2018). Hence, our study is the first to evaluate the properties and acceptance of such a combination of tropical‐fruit wine.

During wine fermentation, the strain of yeast utilized is of key importance as well. So far, *Saccharomyces cerevisiae* has been commonly used in the baking and brewing industries for its excellent properties.

The purpose of this study was to compare the oenological, physical, and chemical components of a novel mixed‐fruit wine (pitaya, watermelon, and mint leaves) with wine produced from pitaya only. The oenological and physical properties analyzed were based on pH, sugar content, alcohol content, color, and specific gravity (SG). The antioxidant abilities of the wines were also analyzed and compared.

## 2 MATERIALS AND METHODS

### 2.1 Materials

Pitaya and watermelon were purchased at a local market in Neipu, Pingtung County, Taiwan. Mint was grown in the Department of Food Science at the National Pingtung University of Science and Technology. Fruits were properly washed, peeled, and cut into cubes. The ratio of pitaya, watermelon, and mint for the mixed‐fruit wine was 50%, 40%, and 10%, respectively. Only pitaya was used for the single‐fruit pitaya wine. All fruit materials were blended and the juice was filtered through a cotton cloth (Gong, Yang, et al., 2017).

### 2.2 Fermentation process

After preparing the juice, the pH and sugar content of the juice were measured. Then, sugar was added to increase the sugar content to 20°Brix. 10 g of *Saccharomyces cerevisiae* (RED STAR®) were then added and allowed to ferment at room temperature for 7–14 days under dark conditions between 25 and 30°C (Gong, Ma, et al., 2017). The fermentation process was stopped using a 0.22 μm filter system (Corning).

### 2.3 pH and sugar content

The pH was determined with a pH meter, and the sugar content of the wine was measured with a Brix refractometer.

### 2.4 Specific gravity

Specific gravity is the ratio of the density of a liquid to the density of water. It means the more the sugar the higher is the SG (Okeke et al., 2015). The following SG formula was used:

(1) $$\text{Specific Gravity}\;\left(\mathrm{SG}\right)=\frac{{}^{\circ}\text{Brix}}{258.6-\left({}^{\circ}\text{Brix}/258.2\right)}\times 227.1+1\;$$

### 2.5 Alcohol content

The alcohol content of the wines was measured using a hydrometer. First, a sample was added to the measuring cylinder. The hydrometer was then inserted, and the reading was taken (Lin et al., 2012).

### 2.6 Color assessment

The color parameters, including L* (lightness of colors), a* (red to green), and b* (blue to yellowness) were measured using a spectrophotometer. The chroma and hue angles were also calculated to express the purity and color of wine according to the following formula (Clemente‐Jimenez et al., 2005):

(2) $$\text{Chroma}=\sqrt{a^{2}+b^{2}}$$

(3) $$\mathrm{Hue}\;\text{angles}=\left[b÷a\right]\tan^{-1}$$

### 2.7 TPC assay

The total phenolic content (TPC) of pitaya and mixed wine was conducted according to the Folin–Ciocalteu method with slight adaptations (Margraf et al., 2015). Firstly, 20 μL of the sample was mixed with 100 μL of Folin reagent (diluted with distilled water at a 1:10 ratio) in a 96‐well microplate. After 5 min, 7.5% sodium was added to the mixture, homogenized, and stored in the dark at room temperature for 30 min. The absorbance was then measured at 765 nm. TPC was calculated from a standard curve of gallic acid. The data was presented as milligrams of gallic acid per 100 g of dried sample.

### 2.8 DPPH assay

The DPPH radical solution was prepared by adding 0.0039 g of DPPH into 100 mL of methanol. A standard solution of butylated hydroxytoluene (BHT) at 6 different concentrations (0, 10, 20, 30, 40, 50 μg/mL) was also prepared. Samples of fresh wine were also prepared at the same concentrations as those of BHT. Ethanol was used as a control. Then, 100 μL of samples at different concentrations (experiment group) were added to 50 μL of DPPH solution in a 96‐well plate where the absorbance was measured at 515 nm. As for the background group, 100 μL of sample at different concentrations was added with 50 μL of ethanol (de Souza et al., 2018). The scavenging ability of DPPH was determined according to the following formula.

(4) $$\text{DPPHs}=\frac{\text{experiment group}-\text{background group}\;}{\text{background}}\times 100$$

### 2.9 FRAP assay

The FRAP assay was carried out by a slightly modified procedure, according to de Souza et al. (2018). A FRAP reagent was freshly prepared before the assay. First, 0.155 g of sodium acetate and 0.8 mL of acetic acid was added to 50 mL of double distilled water (DDW). Then another solution of 0.156 g of 2,4,6‐tripyridyl‐s‐triazine and 2.083 g of hydrochloric acid, were mixed with 50 mL of DDW. Lastly, 0.136 g of ferric trichloride was added to 25 mL DDW. The three solutions were then combined and mixed at a ratio of 10:1:1. In the experimental group, 5 μL of fresh wine sample at different concentrations were added into 15 μL of DDW and 150 μL of FRAP reagent. Absorbance was then measured in a 96‐well plate at 595 nm. In the background group, 5 μL of sample was added to 150 μL of ethanol and 15 μL of DDW.

### 2.10 Sensory evaluation

In this study, the sensory acceptability test for mixed‐fruit wine and pitaya wine was evaluated through hedonic sensory attributes including color, aroma, sweetness, sour, bitter, texture, aftertaste, and overall acceptance. Samples were randomly arranged to minimize bias (Handa et al., 2012).

The scale was divided into 1–9 points according to the degree of preference. The scoring standard applied included the following, 1‐ Dislike extremely, 2‐ Dislike Very Much, 3‐ Dislike Moderately, 4‐ Dislike Slightly, 5‐ Neither Like nor Dislike, 6‐ Like Slightly, 7‐ Like Moderately, 8‐ Like Very Much, 9‐ Like extremely. The sample volume was 20 mL/cup. Water was provided to the participants to rinse their mouths between evaluations. A copy of the evaluation sheet is annexed in Appendix 1. A total of 30 people agreed to do the sensory evaluation after signing an Informed Consent Form (ICF). Out of the 30 questionnaires collected from participants aged 20–24, 56% were from males and 44% from females. However, only 27 of the questionnaires were valid. The selected panelist was evaluated based the completeness of their questionnaires; three of them did not manage to fill the questionnaire properly. Hence, this study ended with 27 valid questionnaires.

### 2.11 Statistical analysis

In‐triplicate experimental results were expressed as means ± standard error (SE) and were statistically analyzed by one‐way analysis of variance (ANOVA) with IBM, SPSS Windows 10. Duncan's multiple‐range test was utilized to compare the significant difference between treatments at 0.05 level. In addition, tables were analyzed by Student's independent *t*‐test.

## 3 RESULTS AND DISCUSSION

### 3.1 Proximate analysis

Pitaya and mixed wines were produced successfully. Sugar content in food plays an important role, especially in the fermentation of fruits. The change of °Brix during the fermentation process is shown in Table 1. The initial and final sugar contents of the mixed wine were 22 and 8 °Brix while those of pitaya wine were 20 and 7 °Brix, respectively (Table 1). Based on the results, the fermentation period was about 5 days which is considerably fast. On days 6 and 7 of the fermentation process, little to no changes were observed which was an indication that the fermentation process had concluded. This concurred with the fact that the alcohol content of the wines reached the maximum between day 6 and 7 as well. Similarly, there was a steady decrease in the density of the wine during the fermentation process. The initial SGs of mixed and pitaya wine were 1.092 and 1.083, respectively. After day 7, the density of the wines decreased to 1.032 and 1.028, respectively (Table 2). According to Xiao Gong et al. (2017), a SG of less than 0.990, is indicative of slow fermentation which might be related to the low concentration of carbohydrates and increased mortality of yeast.

**TABLE 1:** Change in sugar content during the fermentation process of mixed and pitaya wine.

Days	°Brix		
	Mixed wine	Pitaya wine	*p* < .05
0	22 ± 0.001*	20 ± 0.003	.004
1	17 ± 0.024*	18 ± 0.098	.000
2	15 ± 0.018*	17 ± 0.008	.001
3	14 ± 0.098*	13 ± 0.108	.006
4	12 ± 0.002*	11 ± 0.005	.000
5	10 ± 0.000	10 ± 0.001	.090
6	9 ± 0.001	8 ± 0.089	.060
7	8 ± 0.006*	7 ± 0.002	.002

*Note*: The difference between the mixed wine and pitaya wine groups was analyzed using the Student's independent *t*‐test, in which *p* < .05 was considered significant. (*Note*: mixed wine = pitaya fruit, watermelon, and mint).

**TABLE 2:** Change in SG during the fermentation process of mixed and pitaya wine.

Days	Specific gravity		
	Mixed wine	Pitaya wine	*p* < .05
0	1.092 ± 0.0008	1.083 ± 0.005	.003
1	1.070 ± 0.001*	1.074 ± 0.002	.009
2	1.061 ± 0.013*	1.070 ± 0.017	.012
3	1.057 ± 0.007*	1.053 ± 0.009	.001
4	1.048 ± 0.173*	1.044 ± 0.198	.000
5	1.040 ± 0.140	1.040 ± 0.026	1.000
6	1.036 ± 0.000*	1.032 ± 0.001	.008
7	1.032 ± 0.081*	1.028 ± 0.003	.006

*Note*: The difference between the mixed wine and pitaya wine groups was analyzed using the Student's independent *t*‐test, in which *p* < .05 was considered significant. (Note: mixed wine = pitaya fruit, watermelon, and mint).

* means significantly different which is comparing with Pitaya wine.

As evident in Table 3, the pH value of both mixed and pitaya wines slowly increased during the fermentation process. The initial pH values of mixed wine and pitaya wine were 3.15 and 3.35, respectively. After the 7th day of fermentation, their pH values reached 3.44 and 4.19, respectively. The increase in the pH value of wines are directly related to the increase in microorganisms during their fermentation. A similar observation in terms of pH value and changes of pitaya wine during fermentation was reported in a recent study (Xiao Gong et al., 2017).

**TABLE 3:** Change in pH value during the fermentation process of mixed and pitaya wine.

Days	pH value		
	Mixed wine	Pitaya wine	*p* < .05
0	3.15 ± 0.011	3.35 ± 0.009	.061
1	3.16 ± 0.022	3.38 ± 0.011	.093
2	3.24 ± 0.031	3.51 ± 0.005	.055
3	3.28 ± 0.012	3.62 ± 0.008	.082
4	3.33 ± 0.013	3.65 ± 0.012	.094
5	3.35 ± 0.008	3.68 ± 0.003	.050
6	3.42 ± 0.041	3.75 ± 0.021	.092
7	3.44 ± 0.009*	4.19 ± 0.002	.010

*Note*: The difference between the mixed wine and pitaya wine groups was analyzed using the Student's independent *t*‐test, in which *p* < .05 was considered significant. (Note: mixed wine = pitaya fruit, watermelon, and mint).

### 3.2 Antioxidant capacity and TPC

**FIGURE 1:**
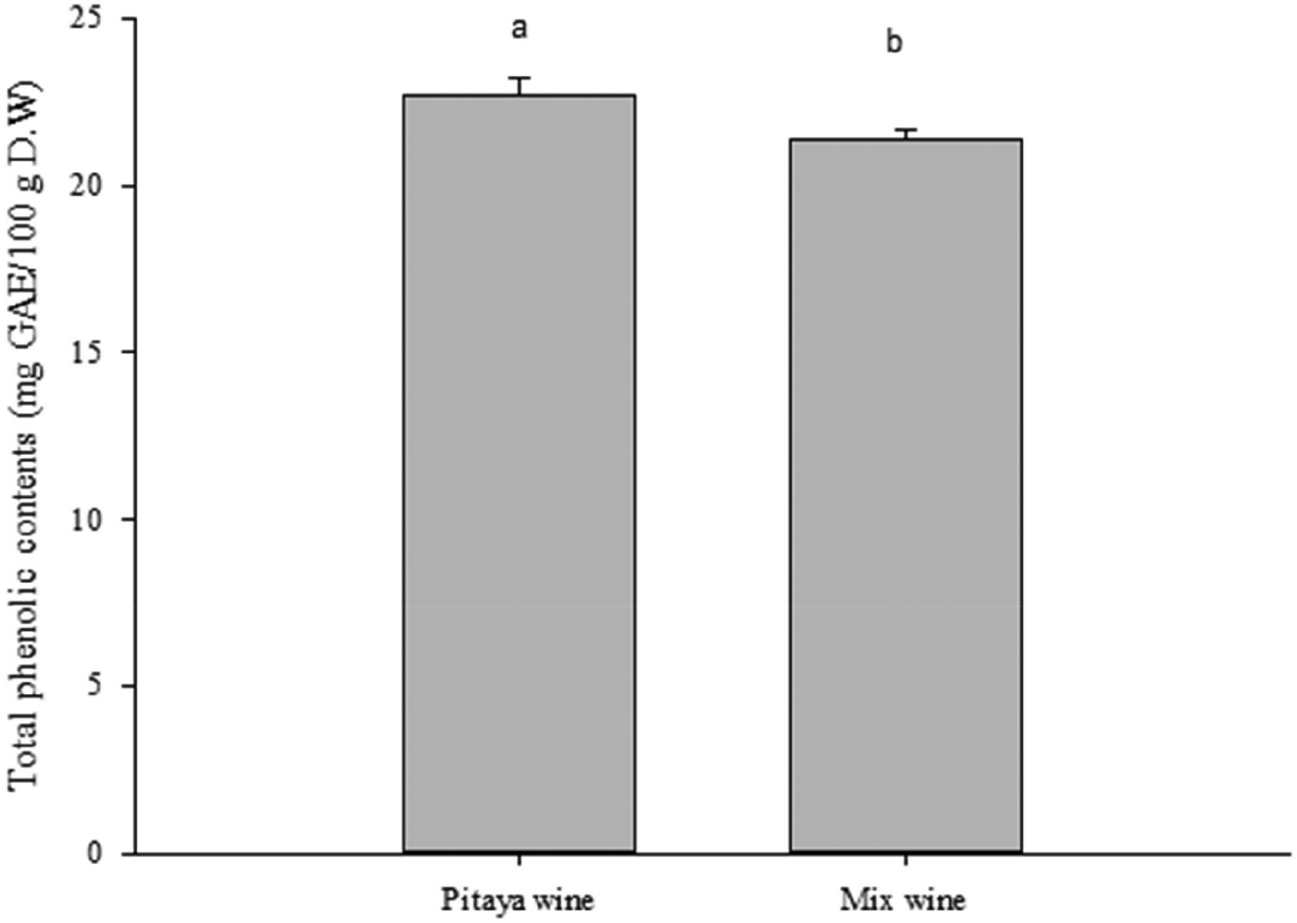
FRAP reducing capacity of mixed wine and pitaya wine. Analysis of variance was performed on in‐triplicate experimental results using SPSS. Duncan's multiple‐range test was used to compare significant difference between groups at *p* < .05 as indicated by different letters.

**FIGURE 2:**
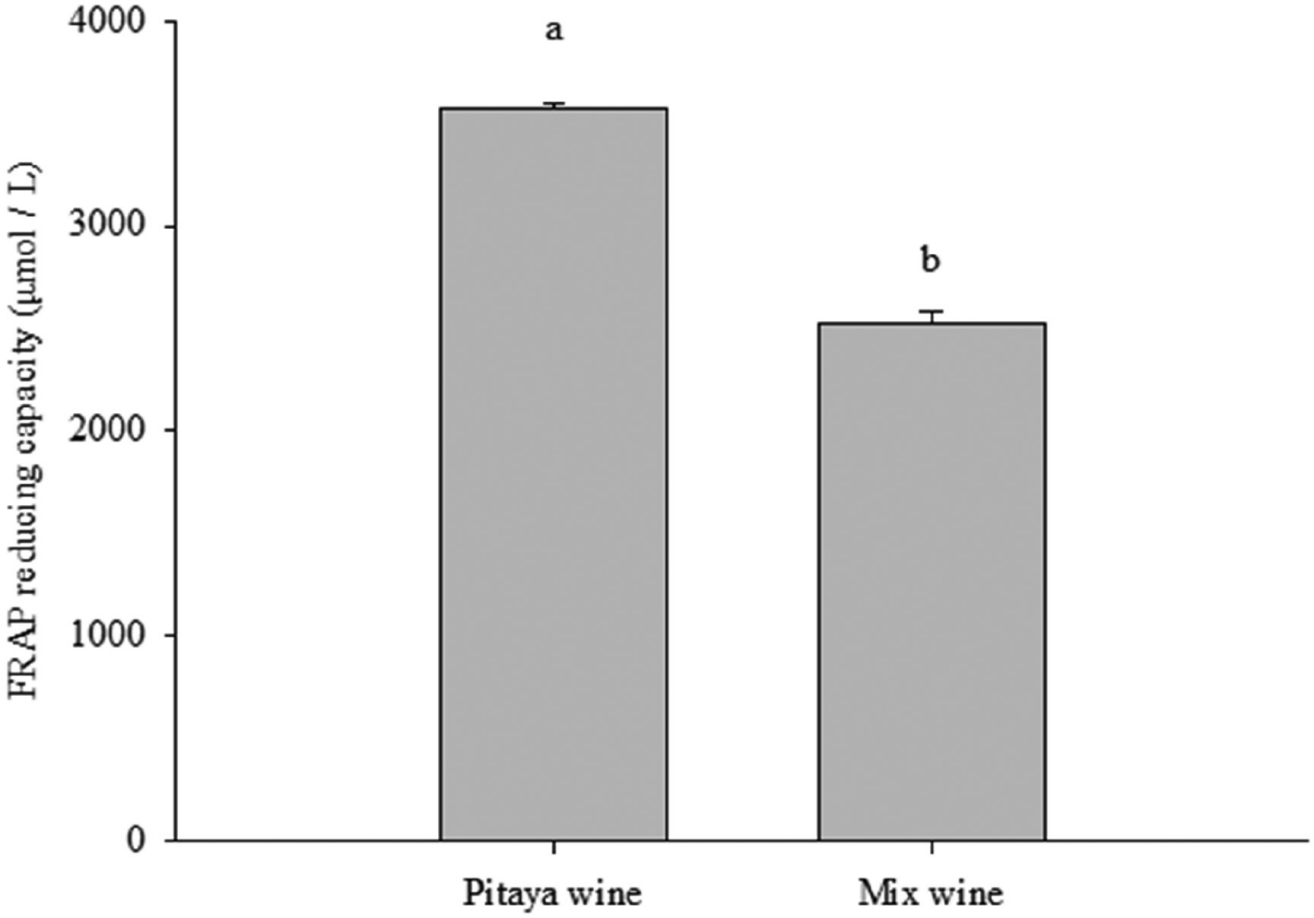
DPPH scavenging activity of mixed wine and pitaya wine. Analysis of variance was performed on in‐triplicate experimental results using SPSS. Duncan's multiple‐range test was used to compare significant difference between groups at *p* < .05 as indicated by different letters.

This study used TPC and radical scavenging tests (FRAP and DPPH) to evaluate the antioxidant activity of mixed and pitaya wines. According to Figure 1, pitaya wine exhibited higher ferrous reducing power (3578 μmole/L) than mixed wine (2528 μmole/L). According to the DPPH assay, the antioxidant activities of both wines were similar to those of FRAP. The pitaya wine showed higher DPPH scavenging activity (80.2%) than the mixed wine (75.6%) (Figure 2). Despite the mixed wine having a lower scavenging ability than the pitaya wine, its antioxidant ability is still acceptable. Our results presented similar results to previous research which indicated that flavonoids, polyphenols, and other substances contained in the pitaya wine may affect its free‐radical scavenging activity (Hui et al., 2021; Yuan et al., 2022). According to Kim et al. (2011), the betanin contents in pitaya also contribute greatly to its antioxidative ability. Another possible reason for the lower radical scavenging activity of the mixed wine is that watermelon contains more water and weaker scavenging compounds like lycopene. For instance, Goderska et al. (2022) reported that beets root which contains higher betanin showed a better antioxidant capacity than those of tomatoes rich in lycopene. Lycopene is also a major pigment in watermelon.

**FIGURE 3:**
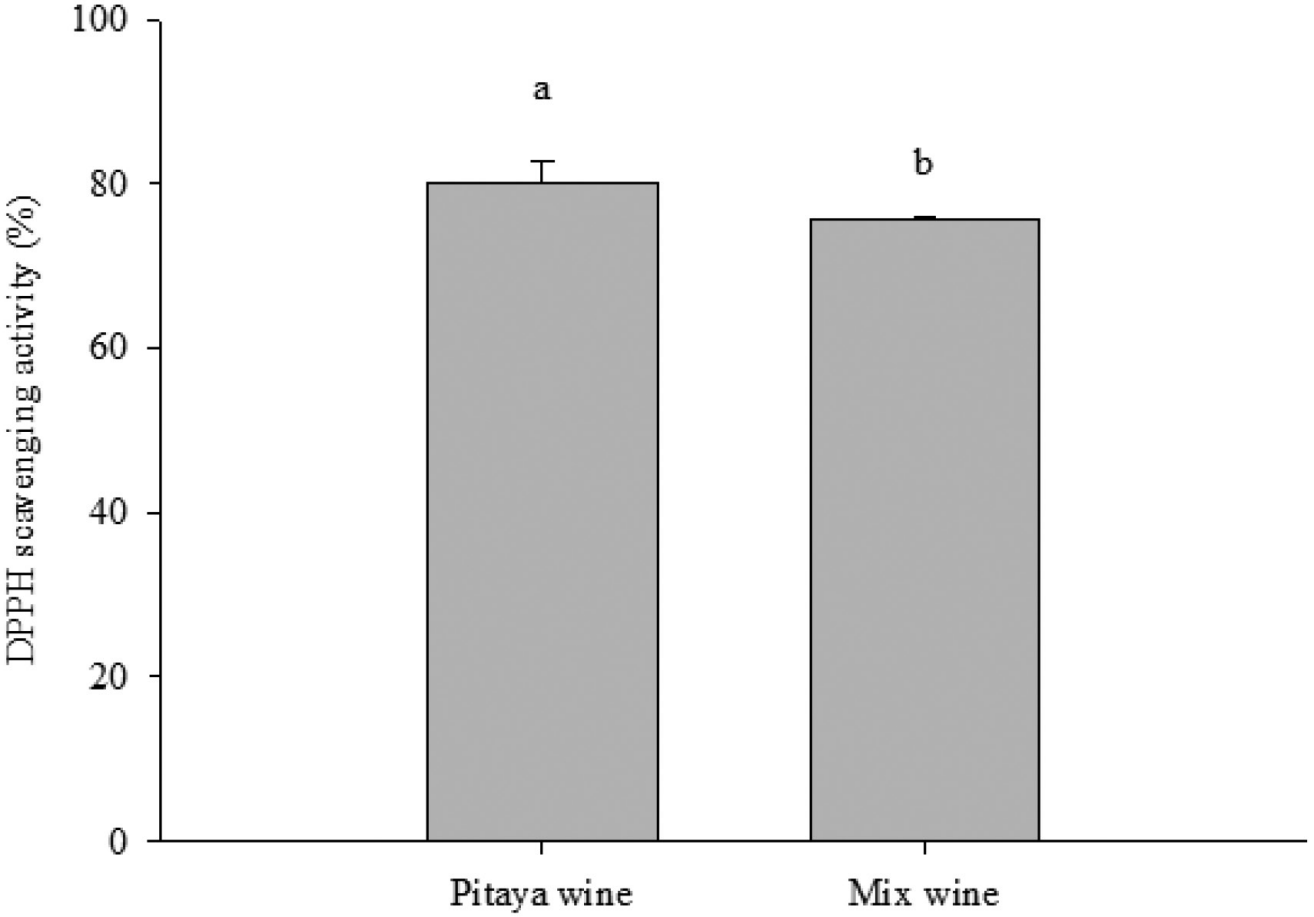
Total phenolic contents of mixed and pitaya wine groups. Analysis of variance was performed on in‐triplicate experimental results using SPSS. Duncan's multiple‐range test was used to compare significant difference between groups at *p* < .05 as indicated by different letters.

In Figure 3, the level of TPCs of pitaya wine is slightly higher than that of mixed wine. Studies have shown that during fermentation, the levels of free phenolic compounds may also decrease (data not shown) through the binding with the molecules in the food matrix, and may be degraded by microbial enzymes and hydrolysis with the specific microbial strains (Melini & Melini, 2021; Othman et al., 2009). Previous studies also presented decreased total phenolic compounds after 8 days of fermentation (Yuan et al., 2022).

### 3.3 Color analysis

Wine color is an important parameter to identify its quality characteristics such as age, type and sort, and composition. Nonetheless, color has been closely linked to pH and temperature. Based on the calculation shown in Table 4, mixed wine (7.49) has a higher chroma value than pitaya wine (6.80). On the other hand, Pitaya wine had a higher hue value (2.86) compared to that of mixed wine (2.29). The incorporation of the chroma and hue values into the color chart indicated that both wines belong under red wine category. This is most likely due to their high content of anthocyanin. Anthocyanins are red pigments that reflect several red–blue hues that are dependent on the pH (Martinsen et al., 2020). Previous research is in accordance with the color findings of this study (Yang et al., 2018).

**TABLE 4:** Color assessment of pitaya wine and mixed wine.

	Pitaya wine	Mixed wine
L*	11.66 ± 0.94	13.06 ± 0.58
a*	−6.80 ± 1.45	−7.49 ± 0.98
b*	−0.35 ± 0.08	−0.35 ± 0.09
Chroma	6.80 ± 0.00	7.49 ± 0.00
Hue angle	2.86 ± 0.00	2.29 ± 0.00

*Note*: The difference between the pitaya wine and mixed wine groups was analyzed using the Student's independent *t*‐test, in which *p* < .05 was considered significant. (Note: mixed wine = pitaya fruit, watermelon, and mint).

### 3.4 Sensory evaluation

**FIGURE 4:**
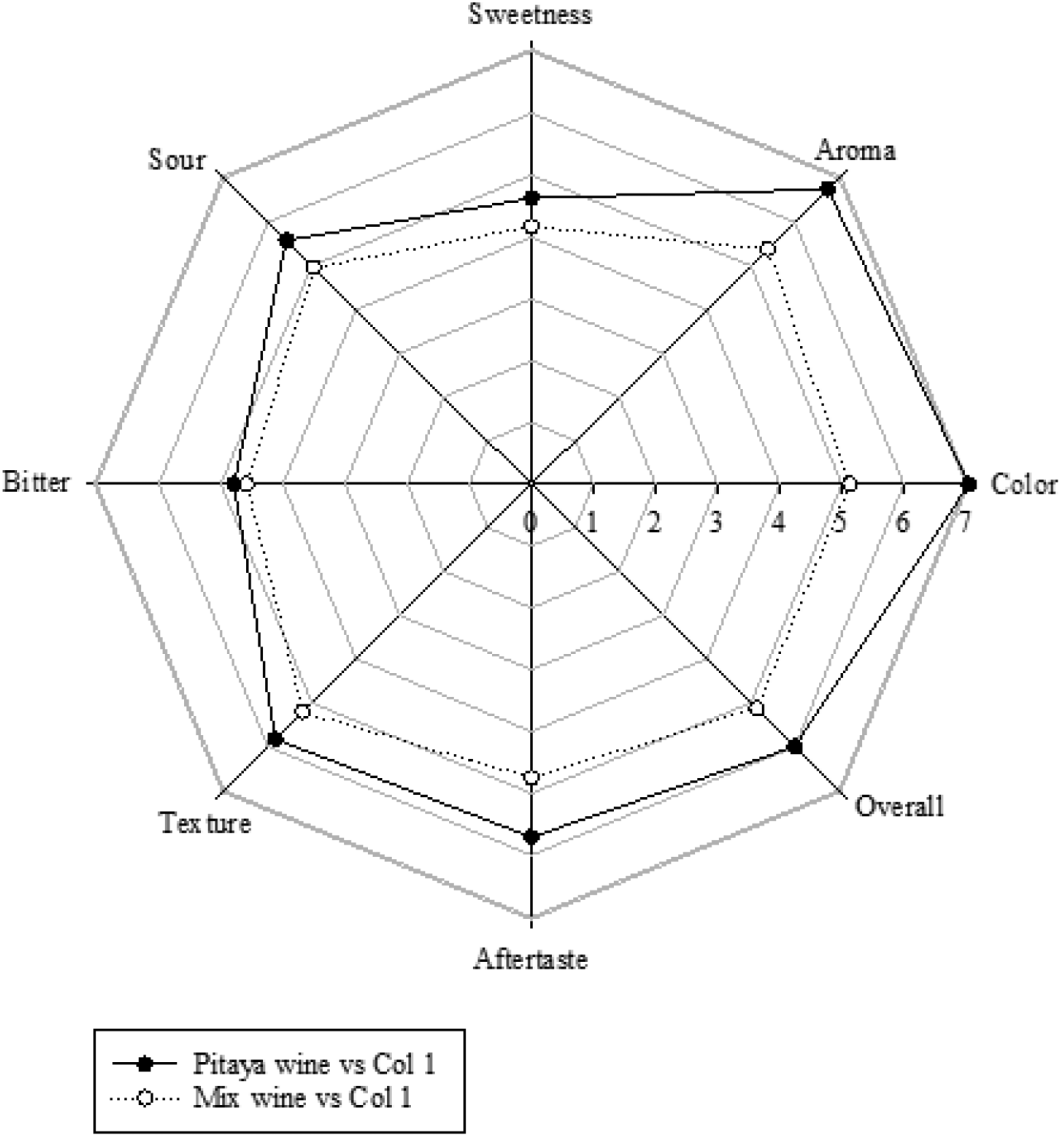
Rose diagram for sensory analysis of mixed and pitaya wines. The selected sensory attributes were color, aroma, sweetness, sour, bitter, texture, aftertaste, and overall acceptance.

Sensory evaluation is of key importance during the innovative development of food and beverage research to investigate consumer acceptability. Results indicate that both pitaya wine and mixed wine received positive scores on all sensory attributes tested, especially on their aroma and color. However, pitaya wine had a better score on all attributes than those of mixed wine (Figure 4). According to some panelists, the strong taste and aroma of mint in the mixed wine did not combine well with the pitaya wine. Such results are similar to a previous study in which the taste of fruit wine was decreased after the wine was fermented with *Lippia javanica* extracts (Chawafambira, 2021). However, there is limited research focusing on the effect on wine quality, hence, it highlights the importance of bioactive and chemical compounds during fermentation (Singh et al., 2021). In addition, some researches have shown that pitaya wine made at 29°C scored the highest for sweetness and spicy senses, but it also produced undesirable odors resulting in the lowest score of global aromas. Hence, it is suggested that wines be produced at 21 to 25°C for optimal flavor properties (Gong, Ma, et al., 2017; Lin et al., 2020). However, the fermentation temperature in this study was between 25 and 30°C, which could be a reason for the unacceptability of some panelists.

## 4 CONCLUSIONS

**FIGURE 5:**
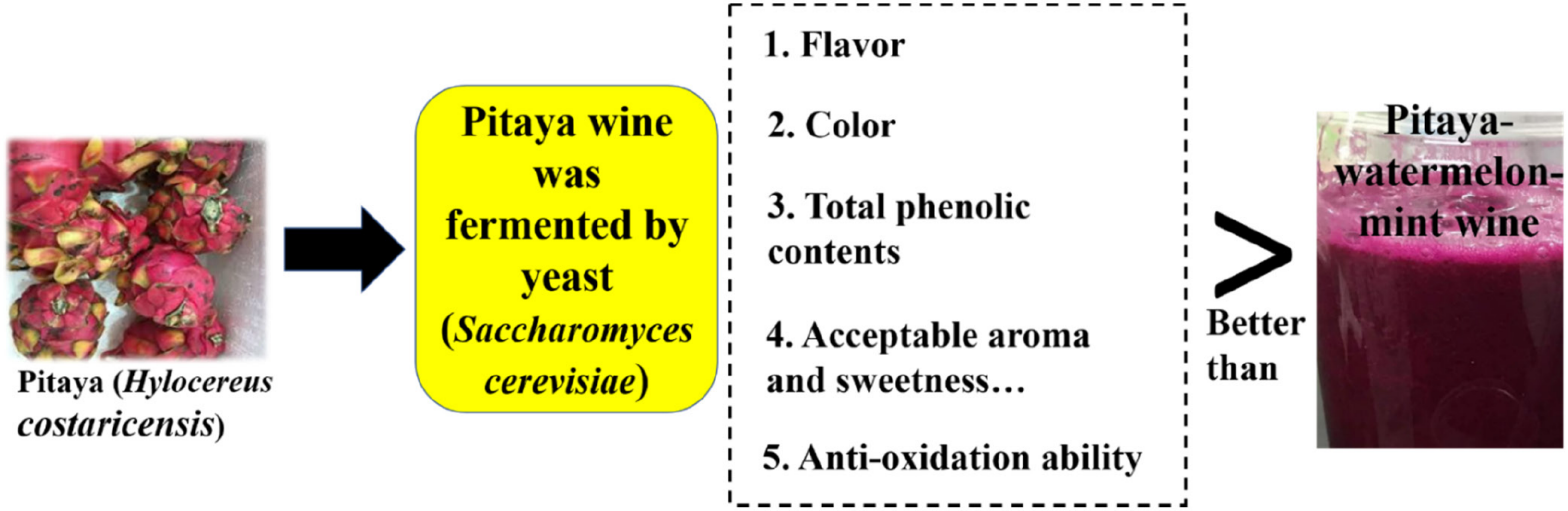
Graphical summary of the experiment.

Results of this research showed that *Saccharomyces cerevisiae* can be used for fermenting mixed wine and pitaya wine without additional sample pretreatment. The amount of yeast and sugar supplemented had a positive influence on the fermentation of the fruits. However, if a higher alcohol percentage is required, other yeast strains should be considered since *S. cerevisiae* can only provide 12% alcohol. At the end of the fermentation, the mixed and pitaya wine contained 11.25% and 11.27% alcohol, respectively. Moreover, the antioxidant capacity was higher in pitaya wine than in the mixed wine. The results indicate that the addition of mint and watermelon did not enhance the antioxidant capabilities or the alcohol percentage of the wines produced (Figure 5). Hence, watermelon and mint may only serve to improve the bitterness of the wines in this study.

## AUTHOR CONTRIBUTIONS

**Tsung‐Ming Hu:** Funding acquisition (lead); supervision (equal). **Mohsen Gavahian:** Investigation (lead); supervision (equal); writing – review and editing (lead). **Rojina Pradhan:** Data curation (lead). **Si Yu Lu:** Data curation (equal); formal analysis (lead). **Yung‐Lin Chu:** Conceptualization (lead); funding acquisition (equal); supervision (lead); writing – original draft (lead).

## CONFLICT OF INTEREST STATEMENT

The authors declare no competing financial interest.

## Data Availability

Data openly available in a public repository that issues datasets with DOIs.

## APPENDIX 1

### 1.1

**Table:**

**Hedonic scale sensory evaluation questionnaire** Age: Gender: Date: **Instruction**: Please taste the following sample from **left to right**, evaluate the color, aroma, sweetness, sour, bitter, texture, aftertaste, and overall by 9‐point scoring method (Integer, could repeat the score). Each sample was 20 mL/ cup. Please evaluate the color and aroma from **top to bottom**, and evaluate the sweetness, sour, bitter, texture, after taste, and overall from **left to right**. Odor dispense is water, please drink the water (or dispense) to remove the aftertaste and wait for **10 seconds** to continue with the next sample.		
Scoring standard 1‐ Dislike extremely, 2‐ Dislike Very Much, 3‐ Dislike Moderately, 4‐ Dislike Slightly, 5‐ Neither Like nor Hate, 6‐ Like Slightly, 7‐ Like Moderately, 8‐ Like Very Much, 9‐ Like extremely		
	259	760
Color		
Aroma		
Sweetness		
Sour		
Bitter		
Texture		
Aftertaste		
Overall		

Suggestions:
